# Taiwanese and Sri Lankan students’ dimensions and discourses of professionalism

**DOI:** 10.1111/medu.13291

**Published:** 2017-04-25

**Authors:** Lynn V Monrouxe, Madawa Chandratilake, Katherine Gosselin, Charlotte E Rees, Ming‐Jung Ho

**Affiliations:** ^1^ Chang Gung Medical Education Research Center (CG‐MERC)Chang Gung Memorial HospitalLinkouTaiwan; ^2^ Faculty of MedicineUniversity of KelaniyaKelaniyaSri Lanka; ^3^ Department of Medical Education and BioethicsNational Taiwan University College of MedicineTaipeiTaiwan; ^4^ Faculty of Medicine, Nursing and Health SciencesMonash UniversityMelbourneVictoriaAustralia

## Abstract

**Context:**

The definition of medical professionalism poses a challenge to global medical educators. This is especially pronounced in settings where professionalism frameworks developed in the west are transferred into different cultures. Building upon our previous study across Western contexts, we examine Taiwanese and Sri Lankan medical students’ conceptualisations of professionalism in terms of *what* professionalism comprises (i.e. dimensions) and *how* it is linguistically framed (i.e. discourses).

**Methods:**

A qualitative group interview study was undertaken comprising 26 group interviews with 135 participants from one Taiwanese (*n* = 64; Years 4–7) and one Sri Lankan medical school (*n* = 71; Years 2–5). Through thematic framework analysis we examined the data for explicit *dimensions* of professionalism. Through *discourse* analysis we identified *how* participants constructed professionalism linguistically (discourses).

**Results:**

Thirteen common dimensions across Taiwanese and Sri Lankan talk were identified, with the dimensions (contextual, integration and internalised self) being identified only in Sri Lankan data. *Professionalism as knowledge* and *patient‐centredness* were dominant dimensions in Taiwan; in Sri Lanka, *attributes of the individual* and *rules* were dominant dimensions. Participants in both countries used four types of discourses previously identified in the literature. Individual and interpersonal discourses were dominant in Taiwanese talk; the collective discourse was dominant in Sri Lankan talk. Findings were compared with our previous data collected in Western contexts.

**Conclusions:**

Despite some overlap in the dimensions and discourses identified across both this and Western studies, Taiwanese and Sri Lankan students’ dominant dimensions and discourses were distinct. We therefore encourage global medical educators to look beyond a one‐size‐fits‐all approach to professionalism, and to recognise the significance of context and culture in conceptualisations of professionalism.

## Introduction

Partly because of high‐profile transgressions of professionalism, public expectations of the medical profession have been changing.^1^, ^2^ As a result, especially in the Western world, the discourse regarding fitness‐to‐practice in medicine has been transformed from one of *competence* (i.e. skills based) to one of *being* a professional (i.e. an embodied identity).^3^ This has led to professionalism in the Western world being an explicit component of education and practice.^4^, ^5^, ^6^ As such, this requires us to understand more fully what comprises professionalism.^7^ This transformation has had a major influence across the world, with many countries adopting Western conceptualisations of professionalism wholesale or with minor modifications.^8^, ^9^, ^10^ Professionalism, however, is increasingly viewed as a socially‐constructed phenomenon: one that is impacted by social, cultural and economic factors.^7^ Thus many believe that the translation of Western professionalism concepts across the world should attend to national cultural differences.^11^, ^12^, ^13^ Our study therefore focuses on medical students’ explicit conceptualisations of professionalism across both Eastern (Taiwan and Sri Lanka) and Western (UK and Australia) cultures.

In this introduction, we will outline key literature on national cultural dimensions, the sensitivity of professionalism to cultural differences and professionalism as conceptualised by medical students and present our study aims and research questions.

### National cultural values

Culture can be defined as a set of attitudes, beliefs, values and self‐definitions shared by a community.^14^ This collective socialisation, manifested in words, thoughts and actions, distinguishes the members of one culture from another.^15^, ^16^, ^17^, ^18^ In terms of a country's culture, whilst acknowledging within‐country differences, there is often strong homogeneity in terms of values amongst members. Further, rather than possessing a unique set of values, different countries demonstrate a complex range of scores (or loading) across similar value clusters.

Numerous theories have been formulated to understand similarities and differences amongst countries’ cultures by identifying broad clusters (or themes) of attitudes, values and beliefs.^14^, ^17^, ^19^, ^20^ In a review of six main theoretical perspectives, Maleki and De Jong identified nine clusters of dimensions: individualism versus collectivism; power distance; uncertainty avoidance; mastery versus harmony; traditionalism versus secularism; indulgence versus restraint; assertiveness versus tenderness; gender egalitarianism; and collaborativeness.^17^ We focus on the three most common clusters identified across all theoretical perspectives.

Individualism versus collectivism characterises the inter‐relatedness of individuals, including aspects such as individual autonomy versus collectivity‐orientation and in‐group loyalty.^17^ This cluster also relates to whether people are judged according to who they are (ascribed attributes) or what they do (achieved attributes). In a culture where ties and relations are prioritised, who one is is more important. *Power distance* recognises that societal power differentials exist, but it is the relative acceptability of hierarchical relations and position‐related roles that is different, especially with regard to members at lower hierarchical levels.^17^ Traditionalism versus secularism relates to long‐term orientation (traditionalism) and relative willingness to change.^21^

These dimensions have obvious implications in the context of students’ understandings of professionalism, their embodied professional identities and their enactment of professionalism. For example, consider the context of witnessing or participating in professionalism transgressions. Students’ cultural beliefs and values around hierarchical relations and in‐group loyalty can impact strongly on their willingness to raise concerns about their seniors, or to comply with or resist seniors’ requests for them to commit a lapse in professionalism.^22^

Different countries have been found to demonstrate a complexity of differences in terms of these clusters.^23^ For example, the UK and Australia have high levels of individualism, whereas there are low and medium levels within Taiwan and Sri Lanka, respectively. The UK and Australia, Taiwan and Sri Lanka have low, medium and high levels, respectively, of power distance (i.e. acceptance of power). Australia, the UK and Sri Lanka, and Taiwan have low, moderate and high scores, respectively, in terms of traditionalism: demonstrating greater resistance to change in Australia, with Taiwan highly orientated towards change.^23^ Indeed, Taiwan has experienced dramatic cultural changes in the past two decades; also, Taiwanese people have recently scored much lower on power distance, bringing them closer to their UK counterparts.^24^

### Sensitivity of professionalism to cultural differences

Against this background of cultural differences, it is unsurprising that conceptualising professionalism as a set of universal principles is challenging.^11^ Indeed, initial discourses have portrayed Western ideas of professionalism as global (e.g. ‘the fundamental and universal principles and values of professionalism’ 25), with other countries adopting similar ideas accordingly. For example, the Accreditation Council for Graduate Medical Education, International Division (ACGME‐I), facilitated the introduction of Western standards, including guidelines for professionalism, into residency programmes in Singapore.^26^ Other Asian countries (including Taiwan) have also referenced ACGME documents in establishing their own domestic accreditation criteria in postgraduate training programmes.^27^ In Sri Lanka, with their British colonial history, the UK seems to influence what professionalism comprises.^28^

Although many non‐Western countries draw on Western professionalism frameworks, numerous studies in non‐Western contexts have found key cultural differences. For example, Ho *et al*.'s framework of professionalism developed by Taiwanese stakeholders of medical education highlights the centrality of different concepts: self‐integrity (underpinned by Taiwan's Confucian philosophical background), and harmonising personal and professional roles.^27^ Furthermore, research undertaken with health care professional groups and academics in an Arabian context included the prominent theme of *dealing with God*, relating to self‐accountability and self‐motivation in an Islamic religious faith.^29^ Finally, research exploring UK, European, North American and Asian medical professionals’ perceptions of essential attributes of medical professionalism found 11 attributes that differed by geographical region: European, Asian and North American doctors emphasised being accessible to patients; being culturally sensitive was only emphasised by North American doctors; being punctual and adaptable to changes in the workplace were highlighted by Asian doctors only; and having the skills to train colleagues was a key feature only for European doctors.^30^

Not only do understandings of professionalism differ according to countries’ cultures, different stakeholder groups diverge around what comprises professionalism: understanding how professionalism is conceptualised by medical students is, therefore, crucial to our understanding of how professionalism is transmitted, understood and ultimately embodied.^31^, ^32^, ^33^

### Professionalism as conceptualised by medical students

Several studies from different countries have explored medical students’ understandings of professionalism.^31^, ^34^, ^35^ For example, Akhund *et al*. 34 asked 193 US, Canadian and Pakistani medical students from a single international medical school to identify the relative importance of professionalism attributes: accountability, altruism, duty, excellence, honesty and integrity, and respect. Interestingly, no significant differences were identified between different demographic groups. However, 60% of the Pakistani participants had experienced basic education originating from Western culture. As such, this may have diluted any cultural differences.

Furthermore, Chiu *et al*.^35^ examined the commitment of 440 Taiwanese medical students from four medical schools to various professionalism items developed from a range of Western and Eastern codes of conduct, including items such as *public health duty* (following the major 2003 SARS outbreak in Taiwan when some doctors avoided their patients). Respondents scored teamwork and interpersonal skills highest, followed by medical knowledge and skills. Less importance was placed on public health duty and protecting patients’ rights.

Finally, Monrouxe *et al*.^31^ examined 200 English, Welsh and Australian medical students’ understandings of professionalism by delineating both *what* they talked about (*dimensions*) and *how* they talked (*discourses*). The researchers identified 19 different dimensions of professionalism in their talk, including competence, teamwork, knowledge, rules, self‐presentation, phronesis and individual attributes. Analysis further identified that these dimensions were constructed within four different discourses: individual, collective, interpersonal and complexity (see Box 1 for definitions).^31^

Box 1 **The four discourses of professionalism^31^**

*Individual*: professionalism as comprising individual characteristics that are held independent of external or contextual influences.

*Collective*: professionalism as applying to a group of individuals who are trusted by society to perform the duties associated with their profession.

*Interpersonal*: professionalism as interactions between individuals participating in an activity.

*Complexity*: professionalism as dynamic and context dependent; a process involving multiple actors and external factors requiring constant renegotiation.

Although research on understanding what medical professionalism means to students across diverse countries such as Taiwan, Pakistan, Australia and the UK has been undertaken,^29^, ^31^, ^32^, ^34^, ^35^ there has been no systematic approach: different studies use different research methods and focus on different research questions. For example, whereas Chui *et al*. 35 and Akhund *et al*.^32^ have shed important light on medical students’ attitudes towards professionalsim, both employed questionnaires drawing on existing Western frameworks rather than asking students to define what medical professionalism meant to them. As such, students’ responses were mapped against existing frameworks. Thus, rather than exploring cultural nuances, they merely reproduced a Western hegemony.^23^, ^24^ What is missing, therefore, is a programmatic understanding of the ways in which medical students across different countries’ cultures define professionalism and how these compare with each other. This is an important issue because findings from such a study could be used to strengthen any challenges to the universality of professionalism and to establish culturally sensitive definitions of professionalism.

Given the cultural similarities and differences across Taiwan and Sri Lanka, this study aims to build on previous research undertaken in Welsh, English and Australian cultures^31^ to address the following unique research questions. RQ1: What dimensions of professionalism and types of discourses can be identified in Taiwanese and Sri Lankan medical students’ definitions of professionalism? RQ2: Which dimensions and discourses are identified as being dominant in Taiwanese and Sri Lankan medical students’ talk? Drawing on data previously collected 31 we address RQ3: Are there any differences in dimensions and discourses identified across Taiwanese, Sri Lankan, Welsh, English and Australian medical students’ talk?

## Methods

### Study design

A cross‐sectional qualitative study using group interviews with a narrative approach was undertaken. A social constructionist perspective was adopted to understand how medical professionalism is conceptualised by medical students through attending to the *whats* (i.e. *professionalism dimensions*) and *hows* (i.e. *professionalism discourses*) identified in their talk.

This study is part of an international research programme examining health care students’ experiences of professionalism dilemmas (situations that students witness or participate in, which they feel to be unprofessional).^36^, ^37^, ^38^, ^39^, ^40^ The first qualitative study with medical students was conducted in Australia, Wales and England;^31^ this second qualitative study has been conducted in Taiwan and Sri Lanka using identical data collection methods.^40^

### Settings

In Taiwan, medical school presently lasts 7 years, with the final 3 years dedicated to full‐time clinical training. In Sri Lanka, medical school is a 5‐year programme with half‐days of clinical training and half‐days of lectures over the third and fourth years and full‐time clinical training in the final year. Final‐year students in Taiwan presently undertake an internship that is similar but not equivalent to postgraduate Year 1 at North American institutions. They take on greater responsibility for patient care than their Sri Lankan counterparts, who play a more observational role.

### Participants

Twenty‐six group interviews were conducted: 12 groups (*n* = 71 participants; 40 female, similar to the school's gender ratio) from Sri Lanka and 14 groups (*n* = 64 participants; 15 female, similar to the school's gender ratio) from Taiwan (total *n* = 135). All participants were native to their country's culture. Participants learning predominately in the clinical context were invited because of our interest specifically in workplace‐based professionalism dilemmas:^36^ thus, Taiwanese participants were in years 4–7 (age range, 20–33 years; mean age, 24.5) of their programme, whereas Sri Lankan participants were in years 2–5 (range, 22–27 years; mean age, 24.5).

### Data collection

Participants at both locations were recruited via advertisements on electronic bulletin boards, as well as through student association representatives. All students read explanatory information and signed the consent form before being interviewed. In the original study (based in Wales, England and Australia) LVM conducted all interviews except one. In the current study, LVM and CER conducted the initial group interviews in Taiwan, with JM (see acknowledgements) and M‐JH as co‐facilitators of the groups, and continuing with the later Taiwanese interviews. Then, M‐JH led the first few groups in Sri Lanka, with MC co‐facilitating and continuing with the later Sri Lankan interviews. All groups therefore followed the same format. In addition to using the same interviewers across studies, we also used the same discussion guide to ensure consistency.^31^ After an ice‐breaking round, the opening question asked participants to explain what the term ‘medical professionalism’ meant to them. Following this, participants were simply asked to ‘tell us about a situation in which you have witnessed or experienced something you think was unprofessional’. Findings in response to this question are reported elsewhere.^40^ This paper focuses only on students’ answers to our opening question about their understandings of professionalism.

All interviews were conducted in English. However, participants were allowed to express themselves in their first language (Mandarin or Sinhalese), alleviating any possible language barriers. With participants’ consent, the group interviews were audio‐recorded, transcribed and anonymised. In total, 31 hours and 32 minutes of data were collected (18 hours and 44 minutes in Taiwan; 12 hours and 48 minutes in Sri Lanka; average interview length of 72 minutes). All data were transcribed and entered into Atlas.ti Version 7.5.2 (Scientific Software Development GmbH, Berlin, Germany) for later coding.

### Data analysis

#### Thematic analysis (to identify dimensions)

Five researchers (LVM, CER, M‐JH, MC and JN) each read a subset of transcripts from the full data and/or listened to a portion of the audio‐recordings concurrently. A primarily deductive approach was undertaken by LVM and CER, who considered the data with respect to the original framework identified earlier.^31^ They were, however, mindful not to force data into predetermined codes so maintained an open mindset sensitive to identifying differences as much as possible. M‐JH, MC and JN approached the analysis primarily from an inductive perspective, as they were less familiar with the original coding framework and had a better understanding of local cultures (Sri Lanka, MC; Taiwan, M‐JH, JN; see acknowledgements). We thus facilitated both inductive and deductive approaches through a team‐based development of the coding framework, with all researchers actively developing the framework. Researchers then came together to discuss and negotiate the coding framework. This process resulted in a number of higher‐order themes and sub‐themes, which were used to code the data by AC (see acknowledgements) and KG. Twenty per cent of the coded data were checked by LVM and disagreements in terms of whether certain excerpts of talk mapped onto specific dimensions and discourses were resolved by consensus, usually by reference to the wider context in which the excerpts were situated.

#### Discourse analysis

A secondary discourse analysis 41 was then undertaken by M‐JH, KG, AC and LVM on the theme around medical students’ explicit definitions of professionalism to identify how students conceptualised the dimensions they highlighted, following the same path as the original study.^31^ Taking the pre‐identified individual, collective, interpersonal and complexity discourses from professionalism policy documents,^31^ the researchers in Taiwan then took each excerpt of participants’ talk assigned to a professionalism dimension and classified them according to one of these four discourses. Data were then charted according to country (Taiwan and Sri Lanka). Following this process, the discourse coding was double‐checked and discussed among researchers to resolve any disagreements in understanding. *Dominant* dimensions and discourses were identified as the most recurrent themes (i.e. those coded with the greatest frequency within our data). These were then compared across the Taiwanese and Sri Lankan datasets. Thus our use of relative quantification in this paper was to facilitate pattern recognition alongside enabling us to verify our qualitative interpretations.^42^

## Results

In this section, in order to answer research questions 1 and 2, we report on medical students’ explicit definitions of professionalism (i.e. professionalism *dimensions*) in Taiwanese and Sri Lankan participants’ talk, as well as the *discourses* within which these dimensions were framed. We examine these in relation to our findings from our previous study to address research question 3.^31^ Data presented to illustrate our findings have been edited for brevity: square brackets indicate words inserted to clarify meaning or to correct mistakes in students’ English. Ellipses indicate words omitted for brevity. A unique participant identifier accompanies each data excerpt, with the first two letters indicating country (SL or TW), the next two indicating year of study (e.g. Y4, Y6), and then gender (F or M) and unique participant number. We offer only one data excerpt per dimension in this paper, but see our online supplement S1 for further illustrations of dimensions and discourses.

### Dimensions of professionalism

We identified 16 dimensions of professionalism across participants’ talk, with 13 dimensions common to both Taiwanese and Sri Lankan data (see Table 1 for patterns mapped against the 19 dimensions identified by Monrouxe *et al*.).^31^ As Table 1 illustrates, the dimensions of *contextual*,* integration* and *internalised self* (defined below) were found in the talk of Sri Lankan participants but not Taiwanese students. Further, the dimensions of *hierarchy* (awareness of one's position and role in hierarchical relationships), *stasis* (having full complement of ethical and professional attributes from the outset) and *self‐care* (legal, physical and emotional protection of oneself) found in Monrouxe *et al*.^31^ were absent from Taiwanese and Sri Lankan participants’ talk. We now define each of the dimensions identified in the current data in turn, providing examples of each from our data.

**Table 1:** Dimensions of professionalism found within participants’ data by country

Dimension	TW	SL	WL	EN	AU
Attributes of the individual	✓	✓	✓	✓	✓
Development	✓	✓	✓	✓	✓
Presentation	✓	✓	✓	✓	✓
Special	✓	✓	✓	✓	✓
Knowledge	✓	✓	✓	✓	✓
Competence	✓	✓	✓	✓	✓
Phronesis	✓	✓	✓	✓	✓
Segregation	✓	✓	✓	✓	✓
Rules	✓	✓	✓	✓	✓
Patient‐centredness	✓	✓	✓	✓	✓
Team‐playing	✓	✓	✓	✓	✓
Role models	✓	✓	✓	✗	✓
Service provision	✓	✓	✓	✗	✓
Integration	✗	✓	✓	✓	✗
Contextual	✗	✓	✓	✓	✓
Internalised self	✗	✓	✓	✓	✓
Hierarchy*	✗	✗	✓	✓	✓
Stasis*	✗	✗	✓	✓	✓
Self‐care*	✗	✗	✓	✓	✓

TW, Taiwan; SL, Sri Lanka; WL, Wales; EN, England; AU, Australia.

*Denotes dimensions.

#### Professionalism as attributes of the individual

Here, professionalism was defined as comprising personal qualities and virtues: for example, being altruistic, capable, a good communicator, maintaining confidentiality, being honest, acting with integrity, being respectful and being responsible. This dimension was found within both Sri Lankan and Taiwanese students’ talk:

[the] way they [doctors] behave … [the] way they move, [the] way they talk, how they deal with people … all are included in … professionalism. (SLY2M4)

Although both groups of participants identified this dimension, it was more dominant in Sri Lankan participants’ definitions, with notions of professionalism as encompassing good behaviours and attitudes and being expressed as primarily comprising personal qualities and virtues. Specific personal qualities, such as ‘dedication’ (SLY3M1), ‘respect’ (SLY5F9) and ‘kindness and empathy’ (SLY5F19) were most commonly highlighted.

#### Professionalism as development

Linked to the notion of individual attributes, professionalism was also defined as a developmental process forming those attitudes, skills, knowledge and behaviours for future professional practice. Again, this was identified in both Sri Lankan and Taiwanese students’ responses. For example, one student defined professionalism as building up one's ‘personality’ (SLY2M2) to make oneself suitable for the role of doctor. Another commented:

I think in the stage of being a student … I think it's more like a long term ability that we have to develop and through real experience too in contact with the patient and in the hospital. (TWY4F30)

#### Professionalism as presentation

Both Sri Lankan and Taiwanese students defined professionalism as a way of presenting oneself to others. As such, it was linked to one's manner of dress, alongside ways of talking and outward actions. This was defined as both the giving of respect to others as well as the requirement to meet others’ expectations:

The respect and other things are coming from the appearance of the person. So we have to dress formal dress code. (SLY5M2)

#### Professionalism as special

Professionalism was also defined as being the exceptional qualities that are attributed to a particular group who hold a position of authority within society. Both groups of students highlighted how merely belonging to this privileged group, or possessing a desire to uphold the reputation of this group, can be reflected in a social contract with those outside the group. For example:

authority … because the doctor has his profession, and is a professional so he has his own knowledge and so sometimes he has the right to decide … maybe the management or treatment of the patient so I believe that it's related to authority. (TWY5M55)

#### Professionalism as knowledge

Professionalism was also defined by Sri Lankan and Taiwanese participants as possessing the knowledge required for professional expertise. It was acknowledged that this requires students’ constant efforts to keep themselves up‐to‐date with the latest medical innovations and information. Although both groups of students defined professionalism in this way, *professionalism as knowledge* dominated Taiwanese participants’ talk, with it being considered as the most important aspect of professionalism:

we should have the medical knowledge that we need … before we go to see the patients, that's a basic. (TWY5F1)

attitude … towards what you're learning right now … you really have to feel the responsibility … to learn as much … as possible … so that you will be capable of handling difficult situations … when you're in the hospital. (TWY4M5)

#### Professionalism as competence

Both groups’ talk contained evidence of *professionalism as competence*: being capable of performing one's job to an appropriate standard in different situations. This dimension also often included the application of knowledge (rather than mere possession of knowledge):

‘I think professionalism is … how to use your knowledge … to help a patient … because we learn a lot of knowledge, but the condition of [the] patient is different case by case.’ (TWY6M5)

#### Professionalism as phronesis

Professionalism as phronesis (i.e. practical wisdom: the ability to negotiate real‐world complexities by applying different combinations of one's knowledge, skills and abilities to particular situations) was present in both Sri Lankan and Taiwanese participants’ talk:

if the person is not able to make the correct decision in that particular situation, his professionalism is somewhat not there … having the capability of taking the correct decisions in that particular situation is very important… (SLY5F12)

#### Professionalism as segregation

Professionalism as a boundary between one's professional and personal lives was identified as being a dimension in both participant groups’ talk. Thus, being careful not to bring personal feelings into the workplace, including notions of what constitutes appropriate behaviours of others (such as overstepping boundaries with patients), was highlighted:

professional life is one thing, personal life is one thing. So you should not jumble it up. So you should wear a dress code to the hospital. You should wear according to the way people expect you to wear. But in other hand you should be smart enough not to interfere with your personal life. (SLY5M6)

#### Professionalism as rules

Professionalism as adherence to explicit and implicit regulations, in particular the expectations of society, was present in both Sri Lankan and Taiwanese participants’ talk:

maybe it's not solely defined by our profession, what our profession is, it's rather on the society. What do they look to us [for]? What do they expect of us when we treat the patients? So their expectations should be the standard of our professionalism. (TWY7M40)

Although present in both groups’ talk, it was particularly dominant in the talk of Sri Lankan students. Indeed, they talked about feeling bound by these rules that came in the form of regulations, codes of ethics, sets of principles and societal expectations. Furthermore, professionalism as *rules, presentation* and *special* appeared to be strongly interlinked. Rules were needed to maintain the divide between doctors and ‘normal people’, and ‘dress codes’ were also part of the rules to be obeyed:

… how we maintain our profession among normal people. In amongst doctors also … how we maintain our behavior within the doctors’ society. And it is not, not just rules and regulations … the way we talk, the way we dress … even our private life, we have to behave in a manner … which protect(s) … ourselves and also our profession …when you are acting in the society I think you should keep [a] mental note in your mind that you are [a] doctor and you should never do something to disgrace your profession. (SLY2F12)

Participants appeared to take rules extremely seriously; there was a strong feeling that patients are vulnerable and in need of help, and that the rules (including ethical rules and dress codes) are key to conveying the message that the doctor is trustworthy and will do the right thing for patients.

#### Professionalism as patient‐centredness

Professionalism as putting patients’ needs above one's own, demonstrating respect, benevolence and compassion and using one's knowledge and abilities to do one's best for the patient was identified in both participant groups’ talk. The concept of patient‐centredness also extended to shared medical decision making with the patient and family members, and health care equality in spite of economic difficulties. Professionalism as *patient‐centredness* was a dominant definition within Taiwanese participants’ conceptualisations. In addition to being more prevalent, Taiwanese participants also seemed to have a more nuanced understanding than did Sri Lankan students, including respecting patients’ lifestyles, the unique relationship that exists between doctors and patients and the understanding that doctors can comfort, even if they cannot cure, their patients:

you should have the passion to take care of people who are suffering. A good doctor should really take … good care … [of] his patients … He will spend more time talking … to them. And he will remember … things in [a] patient's life, such as… who just got married or who [had] … a baby … he will [make the] patient… [the] first priority. (TWY4F2)

you are doctors and they believe in you so, what you can do is, you only can do is just [be with them] but they'll become more maybe more comfortable with my [company]. (TWY6F40)

#### Professionalism as team‐playing

Both Sri Lankan and Taiwanese groups’ talk included an understanding of professionalism as ensuring effective working relationships between oneself and other health care professionals:

Individual behaviour has limited role in hospitals, because it's team work … professionals, collectively provide ultimate services to patient. (SLY5F2)

Taiwanese participants also referred to this in terms of a ‘systems based practice’ (TWY4M20).

#### Professionalism as role models

Professionalism as *role models* was identified in both Sri Lankan and Taiwanese participants’ talk: behaving in a manner that sets an example for their peers and subordinates to either emulate or (in the case of negative role models) learn from:

… when we go into the clinical settings we saw how our teachers or how our senior residents practice, and we see something that is, we think is good, and something we consider that may not [be] as good as we thought. And we will learn from what we saw … in the real world is, I think it's more meaningful. (TWY6M45)

#### Professionalism as service provision

Although being less prolific than other definitions, the idea that professionalism is offering one's medical knowledge and skills as a service to society was present in both Sri Lankan and Taiwanese students’ talk:

it's the commitment to work, to serve people. (TWY5F2)

#### Professionalism as integration

Professionalism as the incorporation of one's personal life into one's professional life, and vice versa, such that one's conduct outside of the workplace impacts one's professionalism was only identified in Sri Lankan participants’ talk:

if he or she is a professional … that has to be there … at home, in the town or family life, as well as in the office or in the workplace. (SLY5M3)

#### Professionalism as contextual

Only Sri Lankan participants identified professionalism as contextual (i.e. behaviour and development influenced by one's environment, including medical culture and local traditions):

it may differ from one profession to another, and responsibility also differs, and [is] not the same for a doctor as … for a manager or some other occupation. (SLY5F31)

#### Professionalism as internalised self

Finally, the notion that professionalism comprises values within oneself, beyond personal attitudes or skills, was only identified within Sri Lankan students’ talk. Further, this dimension was reflected in participants’ discomfort when they or others behaved in an ‘unprofessional’ manner:

… whatever the situation, what I think is a professional should have their own set of principals within the person him or herself. I mean about the way he conduct and how he adapts to a situation … with [his] sense of responsibilities towards the society. (SLY4F3)

### Discourses of professionalism

We now turn to consider the ways in which these dimensions were linguistically framed, with this framing ultimately changing the meaning of the dimension under discussion. For example, being a compassionate person (attribute of individual) can be framed within an individual discourse. However, that same attribute of compassion can be framed within an interactional discourse, where one's compassion is constructed as an interactional, patient‐centred activity. We found that participants framed their definitions of professionalism within all four types of discourses previously identified by Monouxe *et al*.:^31^ individual, collective, interpersonal and complexity (for examples, see Table 2).

**Table 2:** Discourses of professionalism with examples

Type of discourse	Example
Individual (dominant in Taiwan)	‘any professional doctor… what … knowledge … he should have’ (dimension, knowledge; TWY7M15)
Collective (dominant in Sri Lanka)	‘if that person as long as he never forget who he is or his responsibilities or his duties then the value of that profession will be safe’ (dimension, attributes of individuals; SLY5M33)
Interpersonal (dominant in Taiwan)	‘like wearing a formal dress code and white coat it's part of the professionalism (small group soft laughter) … and people see you in white coat … their disease have been improved (group soft laughter) because just seeing you wear white coat, they're confident in you, you show them professionalism, it's a placebo effect’ (dimension, presentation; TWY7M30)
Complexity	‘you have some knowledge background, and some medical skills … [as well as] focus[ing] on patient's disease or illness, you have to consider patient other aspect about the patient … his family, or his social status or economies … and … the interaction between doctor and patient … emphasis [on] the relationship between the doctor and the patient’ (dimension, patient centredness; TWY7M24)

As with the dimensions, certain discourses were dominant across the different groups: in Taiwan, the dominant discourses were interpersonal and individual, whereas the dominant discourse in Sri Lanka was collective. In terms of the individual discourse, Taiwanese participants defined professionalism as individual attributes or internally self‐defined sets of responsibilities:

… you always need to make some decision … but you need to make the decision doing the best to you, and to the patient and to their family. (dimension, patient centredness; individual discourse; TWY4F12).

In addition to the individual discourse, the interpersonal discourse was also commonly used: Taiwanese participants described professionalism in terms of relationships with patients and other health care professionals, citing the importance of cooperation between people:

… the whole medical team, including the nurses and others, such as social workers, to really care [for] … the patient, and not only the patient … [but] also the patient's family. (dimension, patient centredness; interpersonal discourse; TWY4F2).

Sri Lankan participants frequently defined professionalism within a collective discourse, suggesting that professionalism applies to doctors as ‘a particular group of individuals’ with ‘common features’ or ‘common limits’ (dimension, attributes of the individual; collective discourse; SLY5M2). The ‘normal’ people expect that ‘this particular occupation or this particular person should have some added qualities and added features … [compared to] what is … expected from the rest of the people’ (dimension, special; collective discourse; SLY5M2).

## **Discussion**

Our research addressed questions around the dimensions and discourses (and the relative dominance of them) identified in Taiwanese and Sri Lankan medical students’ definitions of professionalism, and compared them with those of students from the UK and Australia.

### Summary of key findings

In terms of our first research question, Taiwanese and Sri Lankan participants defined professionalism using a similar range of dimensions, with *contextual*,* integration* and *internalised self* being identified in the Sri Lankan data only. The dominant dimensions identified in Taiwan were *knowledge* and *patient‐centredness*, whereas the dominant dimensions identified in Sri Lanka were *attributes of the* individual and *rules*. As for our second research question, although both groups made use of all four discourses, the dominant discourses in Taiwan were individual and interpersonal, whereas in Sri Lanka the dominant discourse was collective. In terms of our third research question, our Taiwanese and Sri Lankan findings show some similarity to those found in data from Wales, England and Australia.^31^ Eleven dimensions of professionalism were common to the data from all five countries, and all four types of discourses of professionalism were identified for each country. Additionally, in the data from Sri Lanka, England, Wales and Australia, professionalism as *contextual* was a shared dimension, and professionalism as *rules* was a dominant dimension. However, unlike our earlier UK and Australian data, no participants in Sri Lanka or Taiwan defined professionalism as *self‐care, hierarchy* or *stasis*. Although the individual discourse was not dominant in the Sri Lankan students’ talk, it was dominant in the talk of students from all other countries studied.^31^ These results highlight the multidimensional and context‐sensitive nature of understandings of professionalism. Despite overlaps in students’ understandings of professionalism, our findings suggest that professionalism might mean different things to different people in different countries.

### Discussion of key findings in light of existing theory and literature

Distinctions in professionalism dimensions and discourses across national boundaries might be explained by local cultural factors. For example, the dominant dimensions of *knowledge* and *patient‐centredness* in Taiwanese students’ talk is consistent with previous research findings that Taiwanese students placed relatively high importance on medical knowledge and interpersonal skills,^43^ along with accountability to patients and respect for them and their families.^43^ The Taiwanese education system emphasises Confucian teachings, of which knowledge and humanism are core tenets.^44^, ^45^ Indeed, researchers have observed that patient welfare and patient autonomy are among the most common aspects of medical humanities practised by Taiwanese interns.^46^ In recent years, patient‐centred cultural competence training has been introduced, and might have impacted on students’ conceptions of professionalism.^12^, ^47^

In Sri Lanka, the dominant dimension of professionalism as *rules*, could be partly explained by British colonial and Commonwealth history, as this dimension was also dominant in Welsh, English and Australian data. In Sri Lanka, for example, medical education is considerably influenced by structures and identities imparted during the colonial period.^48^, ^49^, ^50^, ^51^ As such, teaching in Sri Lankan medical schools has tended to place emphasis on proper ethical behaviours, as outlined in various professional codes.^52^, ^53^ Although the dominance of the professionalism dimension of *attributes of the individual* in Sri Lankan students’ definitions might likewise be influenced by colonial ideas, this finding could also be explained by aspects of Sri Lankan culture. For example, Buddhist Sri Lankans, who comprise the majority of the country's population, place great importance on the refinement of personal attributes and proper conduct.^53^, ^54^

The collective discourse was identified in the Sri Lankan data but not in the Taiwanese data, and could be explained by their emphasis on professionalism as *rules*; that is, involving codes of ethics and a social contract that constructs the profession as autonomous from, but in service to, ‘other’ or ‘normal’ members of society. Although Taiwanese students’ talk did not contain substantial use of the collective discourse, the dominance of the interpersonal discourse identified in their definitions might be linked to a collectivist culture in which the self is fundamentally integrated with others.^55^ Despite a trend towards individualism, Taiwanese culture still values relationships and obligations, with strong interpersonal relationships viewed as the key to social stability and harmony.^56^, ^57^, ^58^, ^59^ Previous research has indicated that, in addition to knowledge, Taiwanese students prioritise interpersonal skills and teamwork as dimensions of medical professionalism.^42^ The individual discourse also dominant in Taiwanese students’ talk might indicate a recent shift towards ‘Chinese‐style’ individualism alongside growing Western influence.^60^, ^61^, ^62^, ^63^ Other research on medical professionalism in Taiwan has highlighted individualism, finding that, although students strongly supported patient‐centredness, they did not support commitment to patient care to such an extent that it might reduce their own quality of life.^43^

One interesting omission in Taiwanese and Sri Lankan students’ definitions of professionalism was that neither defined professionalism in terms of hierarchy, which was present in both Welsh and Australian data. Taiwan and Sri Lanka are traditionally viewed as ‘high hierarchy’ cultures compared with the low levels typically characterising Western countries.^15^, ^19^, ^64^, ^65^ However, new generations of Taiwanese people tend to be increasingly reluctant to accept different levels of power.^44^, ^66^ Likewise, in Sri Lanka, hierarchy underscores a social and political order defined by status‐based relations,^67^, ^68^, ^69^, ^70^ but does not appear in students’ definitions of professionalism. Indeed, given that hierarchy remains an important force in both countries’ local and medical cultures, hierarchy might be normalised and therefore not seen as a problem by students. By contrast, in Wales and Australia, students might have mentioned this dimension because of the stark contrast between hierarchical medical culture and relatively egalitarian day‐to‐day life in society.

As for the lack of professionalism as *stasis* in Taiwan and Sri Lanka, it could be that students in the two Eastern contexts did not perceive themselves as having already ‘attained’ professionalism. In Taiwan, for example, Confucian values of humility and constant self‐improvement might explain the absence of stasis in Taiwanese participants’ definitions of professionalism.^71^, ^72^ However, it must be recognised that *stasis* was not a key feature of UK and Australian students’ talk. *Self‐care* was the least‐cited dimension in England and Australia, but its absence in the current study might be related to the collectivist cultures of Sri Lanka and Taiwan, where there is greater focus on others than on the self. Students might have also internalised the value that ‘ideally’ a doctor should be altruistic and prioritise others over self. Finally, professionalism as *contextual* was identified in Sri Lanka, but not Taiwan. This might be explained by resource limitations in the Sri Lankan health care sector,^73^, ^74^ where students may have observed the need to adapt to circumstances by providing the best possible, but not ideal, care to patients.

### Challenges and strengths of the study

Our study has limitations that must be taken into consideration when interpreting our findings. First, as only one medical school in Sri Lanka and Taiwan participated in this study, our results might not be transferable to other medical schools in those countries, or in Asia more broadly. Second, caution is warranted when interpreting our findings as the medical culture in each country may not be representative of other aspects of that country's cultural identity. Finally, dimensions and discourses absent in students’ talk might be a result of sampling issues, so we cannot conclude that their absence in our data means they are absent in the country's culture.

However, our study also has strengths. Overall, this study has much to contribute to global medical educators as they seek to understand and teach medical professionalism in diverse cultural contexts. There has been little research conducted on medical professionalism in non‐Western settings. We are the first, to our knowledge, to conduct a large qualitative study of students’ conceptions of professionalism across two Asian countries. Furthermore, by building iteratively across studies, we have been able to compare and contrast relative findings across contexts in a greater number of countries, including Western countries.^31^ Finally, our team‐based approach to data analysis has provided additional rigour to the analytical process.

### Implications for educational research and practice

Our study demonstrates that medical professionalism is a multidimensional and culture‐sensitive construct and as such has implications for further research. We studied students from just two countries’ cultures so further research should compare medical students’ conceptions of professionalism across a wider variety of country‐culture settings. Further research would also benefit from better understandings of faculty members’ conceptualisations of professionalism in different country‐culture settings.

In terms of educational practice, we believe that these findings should encourage global medical educators to avoid a ‘one‐size‐fits‐all’ approach in their understanding, teaching and assessment of medical professionalism. Instead, we call for conceptualisations of professionalism as dependent upon local culture. Indeed, we encourage students, teachers and medical schools to develop a shared understanding of professionalism within their own cultural context as a starting point for curriculum development and developing the knowledge, attitudes, skills and practices of the nascent professional. With such an understanding, the professional behaviour of tomorrow's doctors can be developed for the benefit of patients and the profession itself.

## Contributions

LVM, MJH, CER and MC developed the study and contributed to data collection. All authors participated in the analysis and interpretation of data. LVM, MC and KG drafted the article. CER and MJ‐H revised the manuscript critically for pertinent intellectual content. The final version was approved for publication by all authors.

## Funding

Ministry of Science and Technology, Taiwan; Association of Medical Education in Europe.

## Conflicts of interest

none.

## Ethical approval

this study was approved by the institutional review committees at the two participating medical schools. These are not named here in order to protect the anonymity of the participating schools.

## Supporting information


**Table S1** Dimensions of professionalism, additional excerpts.
