# Supplementary material for: Taiwanese and Sri Lankan students’ dimensions and discourses of professionalism
Source: Med Educ. 2017 Apr 25;51(7):718–31. doi: 10.1111/medu.13291 (PMC5485011; doi:10.1111/medu.13291)
Supplement: Supplementary file 1 — Table S1 Dimensions of professionalism, additional excerpts. [file MEDU-51-718-s001.docx]

Taiwanese and Sri Lankan students' dimensions and discourses of professionalism

Monrouxe LV, Chandratilake M, Gosselin K, Rees CE, Ho MJ.

Online supplement: additional data for dimensions and discourses

**Table 1: Dimensions of professionalism, additional excerpts**

| Professionalism as attributes of the individual | “*I come up with something the more like personal characteristic like integrity or [being] patient to others*” (TWY4F26) |
| --- | --- |
| Professionalism as development | “*to become a professional, there should be certain qualities … and they should be developed … throughout … time*” (SLY4M1) |
| Professionalism as presentation | “*we have to meet the expectations … of the patients … we try to look like a doctor*” (TWY6F2) |
| Professionalism as special | “*our Sri Lankan society thinks about doctors as … more than [a] profession; as a service … Because people think … ‘these are the people who can help to save our lives’ … So there is a huge respect in the society for doctors … a doctor’s life is … maybe not [as] simple as others*” (SLY2M5) |
| Professionalism as knowledge | "*As medical professionals ... when we are dealing with patients, when we are dealing with communities we have to have the correct knowledge … up-to-date knowledge*" (SLY5F2) |
| Professionalism as competence | "*But when it comes to doctors, they [patients] expect doctor to be very logical and scientific in terms of finding a solution to their [patients] problems … [if not] the relatives of the patient will say* … ‘*they are not up to the standard of that profession’*" (SLY5M3) |
| Professionalism as phronesis | “*we learn a lot of knowledge but the condition of patient is different case by case, and in a dying person with cancer end stage, you may not use aggressive treatment… I'm in [the] oncology department right now and some oncologist was very aggressive no matter what the patient [thought…] some VS* [Visiting Staff] *are very aggressive. They use all their weapons to fight the cancer and ignore the patient's own [wishes], so I think it's our duty to use our knowledge right, in the right way, in the appropriate way*” (TWY6M19) |
| Professionalism as segregation | “*maybe they have to view the connection with the patient, but they cannot over-connected with the patient*” (TWY4M9) |
| Professionalism as rules | “*you behave and act as you think … society expects from a professional*” (SLY5F19)  “*I think as professionals everybody should be bounded by a code of ethics, as well as principles*” (SLY5F3)  “*binding to the rules*” (SLY3F10)  “*Strict adheren[ce] to the ethical code* …” (SLY5F26) |
| Professionalism as patient-centeredness | “*I think, is that there are maybe many things, maybe clinical skills or basic knowledge when in clinical environment, we will meet a real person and problems… we will encounter some problems maybe you are willing to give your patient the best but sometimes maybe due to the medical economics or maybe the limited resources we need to make some critical choices. Maybe I think how to deal with this and how to explain these effect to your patient, maybe it’s more, especially difficult in Taiwan or Chinese community. So, I think how turn these things and how to turn those clinical skills and basic knowledge to benefit your patient and let them feel they are well treated… it’s the core part of professionalism.*” (TWY7M24)  “*I think as a doctor, he must look at all the patients as patients, not discriminate by educational level or … economic level or ... anything*” (SLY2F6)  “…they don’t judge their patient…” (TWY4M35)  “…respect for your patient and doctor relationship…” (TWY7M20) |
| Professionalism as team-playing | “*to cooperate with the whole medical team, including the nurses and others, such as social workers*” (TWY4F2) |
| Professionalism as role models | “*I have some role models … teachers in this faculty … even some house officers … I feel they are very professional, and I take them as examples*” (SLY5F17) |
| Professionalism as service provision | *"[Professionalism] should be considered as [providing] a service to society*" (SLY5F31) |
| Professionalism as integration | “*Even our private life, we have to behave in a manner which protect[s]… ourselves and also our profession*” (SLY4F22) |
| Professionalism as contextual | “*There is a part from tradition. But we are not going to take everything from tradition. We have to be a… we have to add new things*” (SLY4M33) |
| Professionalism as internalised self | “*to become a professional, there should be certain qualities, and they should be within us from the studentship*” ( SLY4M1) |
